# Antimicrobial neuropeptides and their therapeutic potential in vertebrate brain infectious disease

**DOI:** 10.3389/fimmu.2024.1496147

**Published:** 2024-11-15

**Authors:** Xiaoke Li, Kaiqi Chen, Ruonan Liu, Zhaodi Zheng, Xitan Hou

**Affiliations:** ^1^ Institute of Forensic Medicine and Laboratory Medicine, Jining Medical University, Jining, China; ^2^ College of Medical Engineering, Jining Medical University, Jining, China

**Keywords:** antimicrobial neuropeptides, anti-inflammatory neuropeptides, innate immunity, host defense, neuroimmunology

## Abstract

The defense mechanisms of the vertebrate brain against infections are at the forefront of immunological studies. Unlike other body parts, the brain not only fends off pathogenic infections but also minimizes the risk of self-damage from immune cell induced inflammation. Some neuropeptides produced by either nerve or immune cells share remarkable similarities with antimicrobial peptides (AMPs) in terms of size, structure, amino acid composition, amphiphilicity, and net cationic charge. These similarities extend to a wide range of antibacterial activities demonstrated *in vitro*, effectively protecting nerve tissue from microbial threats. This review systematically examines 12 neuropeptides, pituitary adenylate cyclase-activating peptide (PACAP), vasoactive intestinal peptide (VIP), α-melanocyte stimulating hormone (α-MSH), orexin-B (ORXB), ghrelin, substance P (SP), adrenomedullin (AM), calcitonin-gene related peptide (CGRP), urocortin-II (UCN II), neuropeptide Y (NPY), NDA-1, and catestatin (CST), identified for their antimicrobial properties, summarizing their structural features, antimicrobial effectiveness, and action mechanisms. Importantly, the majority of these antimicrobial neuropeptides (9 out of 12) also possess significant anti-inflammatory properties, potentially playing a key role in preserving immune tolerance in various disorders. However, the connection between this anti-inflammatory property and the brain’s infection defense strategy has rarely been explored. Our review suggests that the combined antimicrobial and anti-inflammatory actions of neuropeptides could be integral to the brain’s defense strategy against pathogens, marking an exciting direction for future research.

## 1 Introduction

Currently, the immune response mechanism of the vertebrate brain remains poorly understood (1, 2). Bacterial, fungal or viral infections in brain tissue are extremely rare due to the presence of the blood−brain barrier (BBB), which offers strong defense against blood-borne pathogens (2, 3). The hypothalamus and pituitary stalk are exceptions to the BBB. Infections in this region, however, are highly uncommon. It is possible that the brain possesses an undiscovered layer of immune defense (2).

Recent studies have indicated the potential role of neuropeptides in regulating the immune response and neuroinflammation (4, 5). They have direct anti-infective properties, protecting nerve tissue from microbial invasion (6). AMPs or host defense peptides, are short proteins found in various living organisms (7). AMPs serve as the host’s primary defense against pathogens and possess the ability to stimulate the innate immune response (8). Many neuropeptides share similarities with AMPs in terms of size, structure, amino acid composition, amphiphilicity, and net cationic charge. *In vitro* studies have shown that certain neuropeptides possess antimicrobial activity (9). The presence of this antimicrobial activity suggests its potential involvement in the innate immune response.

Furthermore, specific neuropeptides synthesized by nerve cells (10) (such as astrocytes and sertoli cells) or immune cells (including lymphocytes, neutrophils, and mast cells) exhibit potent anti-inflammatory effects and actively contribute to the regulation of immune tolerance in various immune disorders (11). It has been reported that immune cells possess receptors for neuropeptides, which confirms the involvement of neuropeptides in immune regulation (12). In response to various invasive and inflammatory stimuli, neuropeptides can inhibit the expression of proinflammatory cytokines (11). Furthermore, they can exert immunomodulatory effects by modulating the balance between effector T cells and regulatory T cells, suppressing inflammation, and maintaining immune tolerance (13). These findings highlight neuropeptides as promising therapeutic candidates for treating autoimmune diseases and inflammatory disorders (11).

Recent studies have revealed the potential role of neuropeptides with antimicrobial (14) and anti-inflammatory (13) properties in the brain’s defense against pathogens. In this review, we comprehensively explore the structural properties and antimicrobial activities of neuropeptides, providing a thorough summary of their antimicrobial effects against various microorganisms, including gram-positive bacteria, gram-negative bacteria, fungi, parasites, and viruses. Additionally, we comprehensively evaluated the immunomodulatory activity of these neuropeptides and their therapeutic potential. The selective utilization of the antimicrobial and immunomodulatory properties of these neuropeptides holds promise for developing a potential therapeutic approach, offering a novel and effective treatment strategy for CNS infectious disease (11).

## 2 Antimicrobial mechanism of AMPs

Antimicrobial neuropeptides represent a unique class of AMPs that possess both neural and antimicrobial properties (1). In this section, we mainly introduce the structural characteristics and antimicrobial mechanism of AMPs to enhance our understanding of antimicrobial neuropeptides.

### 2.1 Structural characteristics of AMPs

The structural diversity of AMPs allows them to adopt different secondary structures, enabling them to employ unique mechanisms for targeting pathogens (15). AMPs exhibit structural variability that is mainly determined by the cell of the peptide source (16). Understanding the structural characteristics of AMPs is essential for further investigation into their antimicrobial mechanisms. Typically, AMPs consist of 10-50 amino acids and have a molecular weight less than 10 kDa (17). The antimicrobial activity of AMPs is influenced by various physicochemical properties, including amino acid composition, peptide length, presence of positively charged residues, lipid composition, hydrophobic characteristics, net molecular charge, and helicity of spatial structure (18).

**Figure 1:**
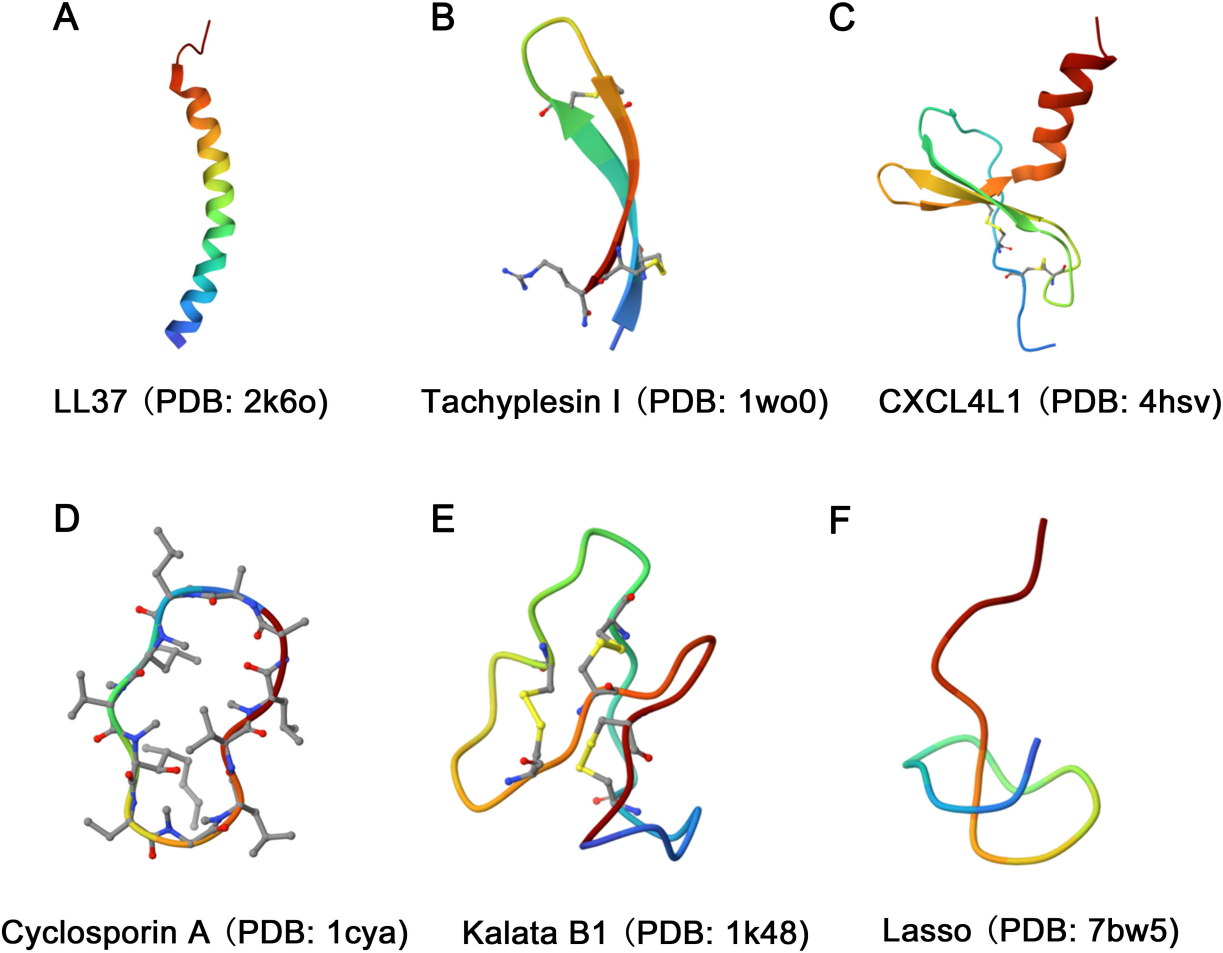
Diversity in the structural characteristics of AMPs. **(A)** The structure of LL37 (PDB ID: 2k6o) is characterized by an α-helix conformation. **(B)** Tachyplesin I (PDB ID: 1wo0) has a typical β-sheet conformation. **(C)** The structure of CXCL4L1 (PDB ID: 4hsv) is classified as an αβ family. **(D, E)** The structures of Cyclosporin A (PDB ID: 1cya) and Kalata B1 (PDB ID: 1k48) were characterized as loop. **(F)** The Lasso (PDB ID: 7bw5) structure exhibits random coiling.

According to the structural model of nearly 900 AMPs, natural AMPs can be classified into four major families: α, β, αβ, and non-αβ (**Figure 1**) (19). Among these families, α-helix and β-sheet structures are the most commonly observed in AMPs found in nature.

The α family of AMPs primarily adopts a linear α-helix conformation as their dominant secondary structure (**Figure 1A**). However, it is important to note that both His-rich and Trp-rich peptides have the potential to form α-helix structures, causing some overlap between classes. Examples of α family AMPs include lactoferricin B, human antimicrobial peptide LL-37, and pituitary adenylate cyclase-activating peptide (PACAP), among others. The β family is characterized by the presence of at least two β chains arranged in a specific structural pattern (20), with cysteine stability and β-folding (**Figure 1B**). Human α-defensins and tachyplesin I are examples of AMPs that adopt this structure. The αβ family of AMPs includes both α-helical and β-sheet conformations (**Figure 1C**). Relevant examples include β-defensins, β-Amyloid (Aβ), CXCL4L1, antimicrobial chemokines, and RNases. Finally, the non-αβ family of AMPs lacks both α-helix and β-sheet structures. However, this family exhibits an extensive secondary structure, including loop peptides (**Figures 1D, E**) and random coils (**Figure 1F**) (21).

### 2.2 Antimicrobial mechanism of AMPs

**Figure 2:**
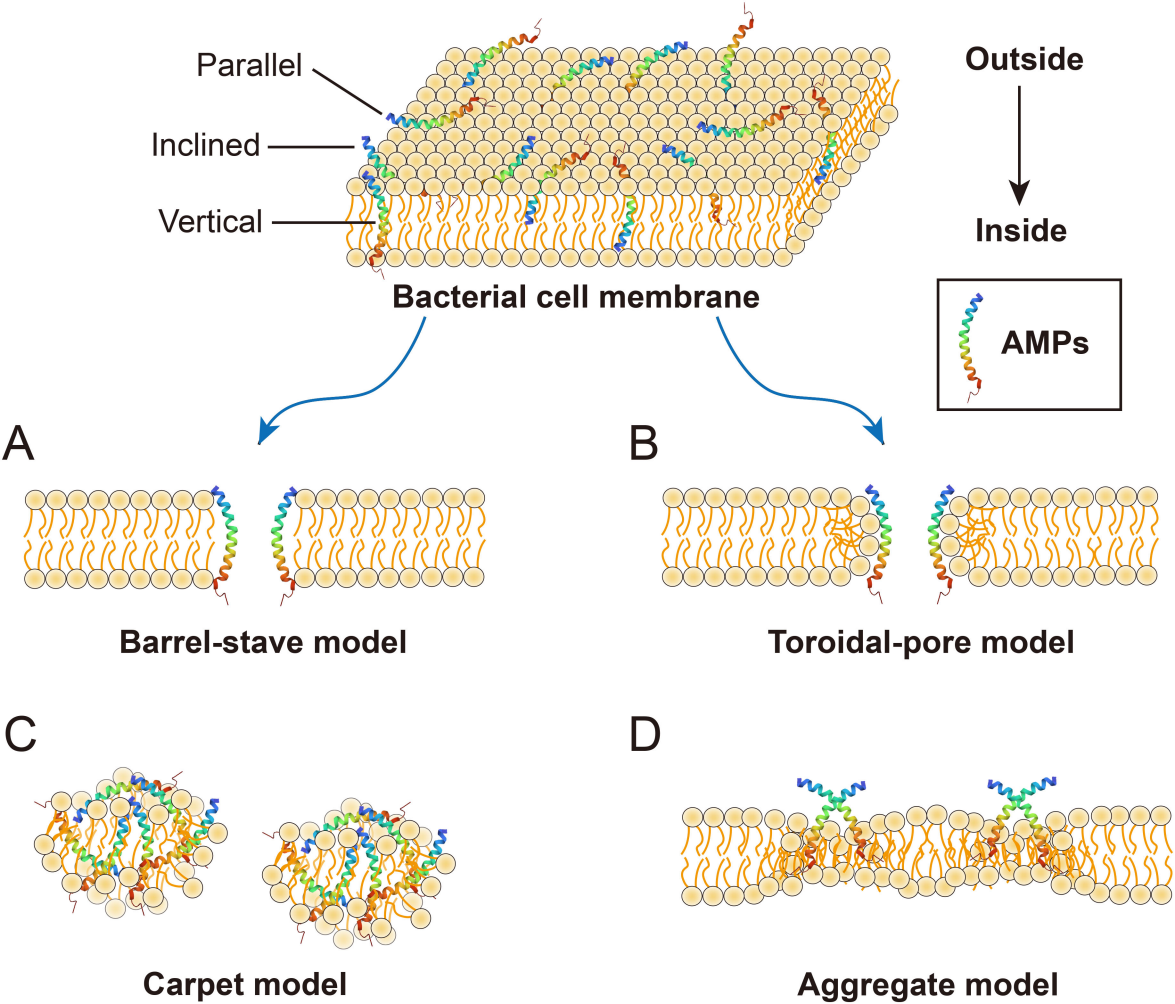
Membrane damage models of AMPs. **(A)** Barrel-septal mode: multiple helical molecules are arranged in parallel to form the central lumen; **(B)** Toroldal-pore mode: it is similar to the Barrel-septal model except that toroidal pore complexes do not need to span the full double layer; **(C)** The carpet model peptide: AMPs cover the membrane in clusters and cause membrane rupture in a surfactant-like manner; **(D)** Aggregate mode: this pattern facilitates the formation of channels, enabling the leakage of ions and intracellular contents, thereby inducing cell death.

AMPs are vital components of the innate immune system and possess strong antibacterial, antifungal, antiparasitic, and antiviral activities (22). Moreover, AMPs play a crucial role in various intracellular processes, such as angiogenesis, arteriogenesis, inflammatory response modulation, cell signal transduction, and the wound healing cascade (23). Numerous mechanisms of action for AMPs have been proposed, but the primary mode of action for most AMPs is the destruction of pathogenic microorganisms by damaging their cell membranes, also known as membrane damage mechanism (24). Antimicrobial neuropeptides also firstly employ this mechanism, similar to that of conventional cationic AMPs, to combat microorganisms. The process can be summarized as follows: (1) Positively charged neuropeptides bind to the negatively charged surfaces of microbes through electrostatic interactions; (2) This binding destabilizes the negatively charged phospholipid bilayer, leading to membrane damage; (3) Membrane permeability is altered; and (4) Microorganisms die due to hypotonicit (25). The membrane damage mechanism of AMPs primarily includes four types: Barrel-stave mode, Toroidal-pore mode, Carpet mode and Aggregate mode. The Barrel-stave model (**Figure 2A**) (26) begins with the incorporation of AMPs into the phospholipid bilayer in three possible orientations: parallel, vertical, or inclined. When the peptide/lipid ratio reaches a certain threshold, resulting in energetical and physical changes in the membrane structure, including the helical hydrophobic regions of the peptides are close to the hydrophobic regions of the membrane phospholipid, while the hydrophilic regions of the peptide are inwards (27, 28). This alignment of helical molecules generated a central lumen, establishing the Barrel-stave model. The Toroidal-pore model (**Figure 2B**) is similar to a transmembrane ion channel and induces bending of the phospholipid bilayer when AMPs accumulate to a certain level. The peptides spiral into the membrane, bind to the lipids, and form a porous ring complex, eliminating the need to span the complete phospholipid bilayer (28). The Carpet model, similar to that of detergent (**Figure 2C**), involves the continuous accumulation of AMPs. When their concentration reaches a threshold, clusters of AMPs cover the phospholipid bilayer, resulting in membrane disruption akin to detergent action without channel formation (18). The Aggregate model (**Figure 2D**) facilitates the formation of peptide-lipid complexes, ultimately leading to ion leakage channels and cellular death (18). The permeability of peptides across membranes is directly influenced by their topological amphiphilic structure rather than by their linear structure, as demonstrated by numerous studies. As a result, the charge is systematically distributed in regular clusters across the polypeptide surface, which forms the basis of its antimicrobial efficacy (29).

**Figure 3:**
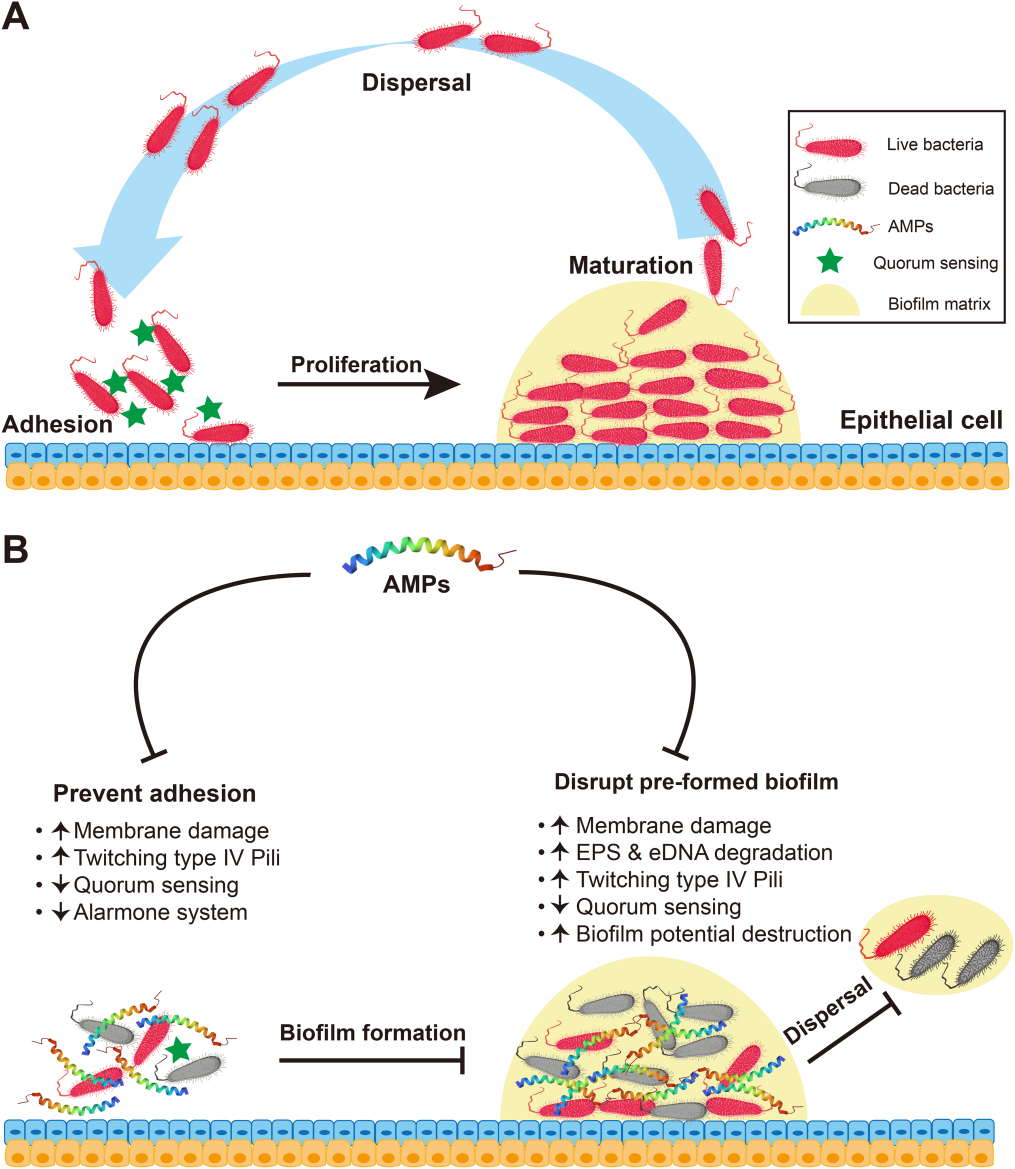
Antibiofilm mechanism of AMPs. **(A)** The process of biofilm formation involves the stages of adhesion, proliferation, maturation and dispersal. **(B)** Antibiofilm mechanism of AMPs. On the one hand, in the inhibition of bacterial adhesion and biofilm formation, AMPs can directly kill preattached pathogenic microorganisms via membrane damage; enhance type IV pili-mediated pulling movement, thereby accelerating bacterial twisting on surfaces and effectively inhibiting bacterial adhesion and biofilm formation; down-regulating the transcription of genes associated with quorum sensing (e.g., *Las*, *Rhl*); and suppress the alarm system to avoid biofilm resistance to AMPs. On the other hand, AMPs can kill preformed biofilms. In addition to causing membrane damage, enhancing type IV pili-mediated pulling movement and down-regulating the quorum sensing, AMPs can enhance the degradation of the synthetic components of biofilm EPS and eDNA and lead to the destruction or degradation of the membrane potential enclosed by a biofilm.

Currently, there is a growing emphasis on investigating the impact of AMPs in inhibiting or eradicating biofilms (30). Unlike phospholipid bilayer membranes, biofilms exist in a rootless form in nature (31). In 1999, Costerton et al. (32) introduced the concept of biofilms, which are structural communities of bacteria, fungi, and viruses adhered to any biotic surface enveloped by a self-produced polymer matrix consisting of proteins, exopolysaccharides, DNA, lipids, and other fragments. In general, a biofilm is an assemblage of organisms formed by the aggregation of microbial cells and matrix (33). The typical biofilm formation process primarily includes four sequential stages: adhesion, proliferation, maturation, and dispersal (34, 35) (**Figure 3A**). Initially, bacteria adhere to the surface, and as their population proliferates, they secrete extracellular polysaccharides, thereby establishing a robust biofilm matrix. Subsequently, the cells continue to grow and divide, facilitating the subsequent detachment and dissemination of the bacteria (36). The effects of AMPs on biofilms primarily include inhibiting the formation and adhesion of biofilms, eradicating preformed biofilms, and impeding biofilm propagation and detachment. However, the mechanism of action of AMPs on biofilms varies across different periods and can be categorized into the following five hypothetical mechanisms (**Figure 3B**): (1) The rapid destruction of biofilm-embedded cells indicates that AMPs act by membrane damage of the bacteria (34). (2) Disruption of quorum sensing signaling: AMPs increase the twisting movement of bacteria on the surface of the biofilm by stimulating type IV Pili-mediated pulling motion, down-regulating the transcription of Las and Rhl in the induction system (37), and down-regulating the genes that migrate and transport binding proteins from extrachromosomal elements to inhibit transporter expression (38), thereby repressing the formation of communal biofilms (34). (3) Repression of the alarm system to mitigate biofilm resistance against AMPs, thereby preventing strict bacterial response (39). (4) Destruction of biofilm potential: after the release of bacteriocin, which disrupts the biofilm matrix, ATP is released, thereby enhancing the permeability of the biofilm and eradicating preexisting biofilms, ultimately leading to bacterial cell death (40). (5) Degradation of the polysaccharide and biofilm matrix. Certain enzymatically active AMPs, such as piscidin-3 exhibit nucleosidase activity capable of impairing extracellular DNA (eDNA) of *Pseudomonas aeruginosa* (39). Peptide PI can degrade the extracellular polymeric substances (EPS) produced by *Streptococcus mutans*, leading to reduced biofilm formation (34). Furthermore, certain non-enzymatically active AMPs, such as hepcidin 20, exhibit the ability to modulate the extracellular matrix integrity by specifically targeting polysaccharide intercellular adhesin (PIA) and inducing structural alterations within the biofilm (34).

In addition to membrane damage and antibiofilm mechanisms, AMPs can also exert their antimicrobial effects through various pathways. First, AMPs can regulate the expression of genes involved in cell wall synthesis, thereby inhibiting this process and exhibiting antibacterial activity (41). Furthermore, AMPs can target peptidoglycan, which is the primary constituent of the bacterial cell wall (42). After the inwards growth of the cell wall and the formation of a transverse cross wall (septum), the newly synthesized peptidoglycan undergoes hydrolysis during cell division under the action of AMP, ultimately resulting in bacterial death (42). Additionally, AMPs possess endotoxin-neutralizing properties that enhance innate immunity and effectively exert antimicrobial effects (43). Finally, AMPs exert their antimicrobial effect by impeding or terminating the translation process, thereby inhibiting intracellular nucleic acid and protein synthesis through complex mechanisms (44–46).

Compared to conventional antibiotics that only target a single site, AMPs possess multiple targets, enabling them to eliminate pathogens from various directions, thereby significantly reducing the emergence of antibiotic-resistant bacteria (47). Resistance to AMPs is more difficult than resistance to antibiotics, and the therapeutic mechanisms employed against drug-resistant bacterial infections can be categorized into the following five different approaches: biofilm penetration, re-sensitization, intracellular bacteriostatic function, immune activity regulation, and biofilm inhibition (47).

## 3 Neuropeptides with antimicrobial activity

**Figure 4:**
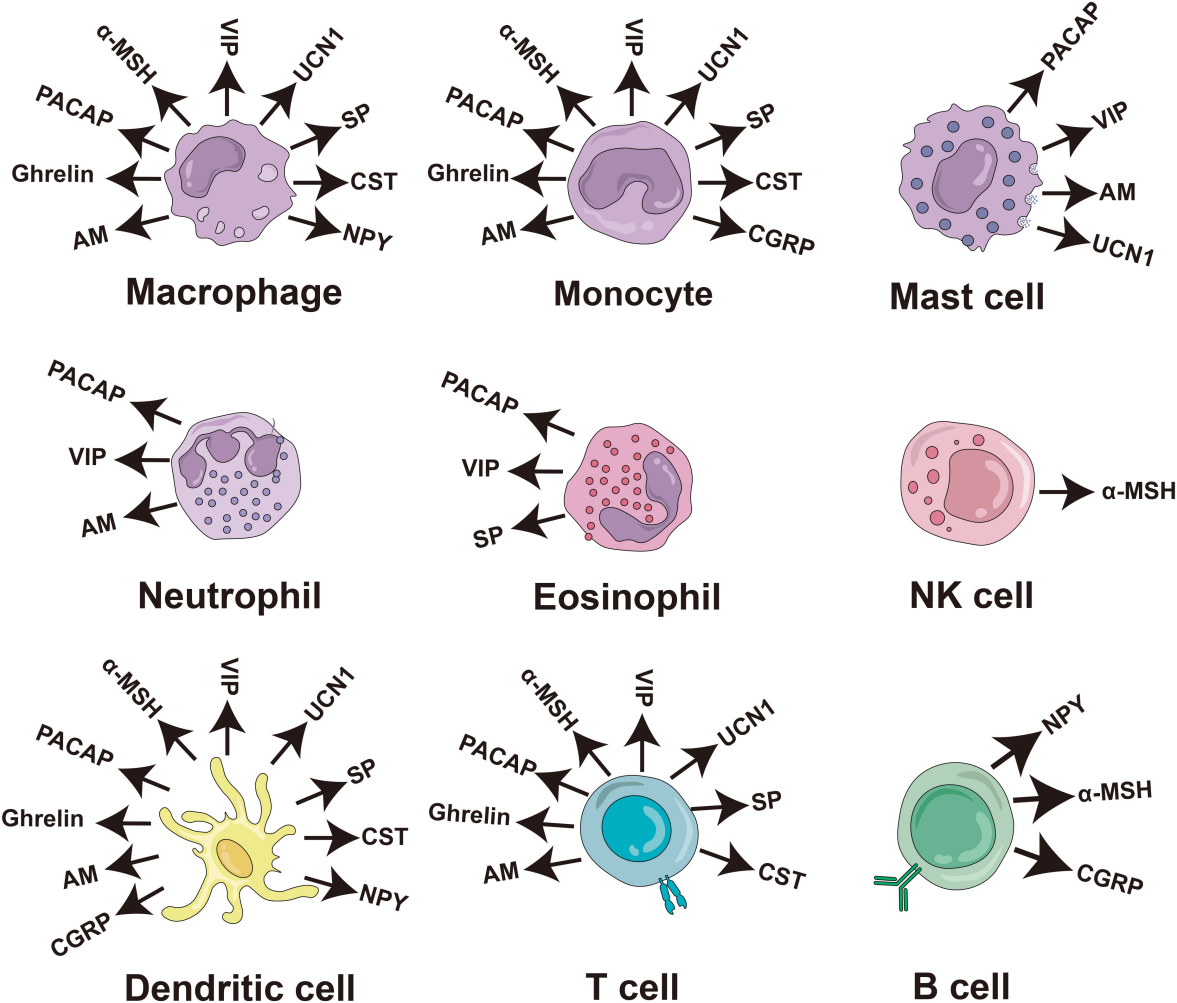
Production of antimicrobial neuropeptides by cells of the human immune system. The diagram of the immune cells was refined based on Krause’s work (48). PACAP, pituitary adenylate cyclase-activating peptide; VIP, vasoactive intestinal peptide; α-MSH, α-melanocyte stimulating hormone; SP, substance P; AM, adrenomedullin; CGRP, calciton-in-gene related peptide; UCN II, urocortin-II; NPY, neuropeptide Y; CST, catestatin; NK cell, natural killer cell.

Numerous studies have consistently demonstrated the direct antimicrobial effects of neuropeptides *in vitro*, substantiating their established role as antimicrobial agents. These neuropeptides include PACAP, vasoactive intestinal peptide (VIP), α-melanocyte stimulating hormone (α-MSH), orexin-B (ORXB), ghrelin, substance P (SP), adrenomedullin (AM), calcitonin-gene related peptide (CGRP), urocortin-II (UCN II), neuropeptide Y (NPY), NDA-1, and catestatin (CST) (**Table 1**). In addition, most of them can be secreted by immune cells and play an immunomodulatory role (**Figure 4**).

**Table 1:** Physicochemical characteristics of antimicrobial neuropeptides.

Name	Length	Charge	GRAVY	PI	Structure	Activity	Inflamm. Resp.	Ref.
PACAP	38	11	-1.06	10.77	α	G, F, V	Anti-inflamm.	(1, 49–52)
VIP	28	4	-0.64	10.20	27% α, 27.2% β, 45.8% RC	G, F, P	Anti-inflamm.	(53–56)
α-MSH	13	1	-0.92	9.72	Unknown	G, F, V	Anti-inflamm.	(57–61)
ORXB	28	4.1	0.78	7.88	α	G, V	Unknown	(62–66)
Ghrelin	28	5.5	1.68	11.53	α	G^-^, P	Anti-inflamm.	(67–71)
SP	11	3	1.16	9.72	α	G, F, V, P	Pro-inflamm.	(72–75)
AM	52	52	-0.89	9.97	α	G	Anti-inflamm.	(76–79)
CGRP	37	4	0.21	9.91	α	G^-^, F	Anti-inflamm.	(55, 80–85)
UCN II	38	4.21	0.53	12.23	α	G, P	Anti-inflamm.	(86–90)
NPY	36	1	-1.19	7.55	α or PP-fold	G, F, V, P	Anti-inflamm.	(91–94)
NDA-1	38	8	-0.82	12.41	β	G, F	Unknown	(95)
CST	21	4	-0.49	12.31	β	G, F	Anti-inflamm.	(96–99)

Charge, net charge; GRAVY, grand average of hydropathy; PI, protein isoelectric point; Structure, the secondary structure of AMPs; Inflamm. Resp., inflammatory response; α, α-helix; β, β-sheet; RC, random coil; PP-fold, consists of a long N-terminal polyproline helix followed by a type II β-bend and a long amphiphilic α-helix; G, Gram positive and negative bacteria; G^-^, Gram negative bacteria; F, fungi; V, virus; P, parasites.

### 3.1 Pituitary adenylate cyclase activating peptide (PACAP)

In 1989, Miyata et al. (100) successfully isolated PACAP from sheep hypothalamic tissue and searched for peptides that can stimulate the secretion of pituitary hormones. PACAP, an α-helical peptide, belongs to the VIP/secretin/glucagon superfamily and exhibits 68% sequence similarity with VIP (51). PACAP exists in two amidated forms, PACAP27 and PACAP38, with PACAP38 being the predominant form (101). It is mainly found in thymocytes, lymphocytes, and plasma cells of the spleen and lymph nodes in the immune system (102), and it has potent anti-inflammatory effects (49).

In 2021, Lee et al. (1) conducted a comparative study of the human neuropeptide and AMP databases using bioinformatics methods. They discovered that PACAP38 showed potential as an AMP, sharing similarities with the known AMP LL37 in terms of secondary structure, amino acid composition, and average hydrophobicity. Subsequently, Lee et al. (1) employed X-ray scattering techniques to demonstrate that PACAP38 can penetrate the bacterial plasma membrane, causing membrane damage. However, it had no effect on mammalian cell membranes. Notably, PACAP38 exhibited significant antimicrobial activity against the gram-positive bacterium *Bacillus subtilis* (minimum inhibitory concentration, MIC: 5 µM), the fungus *Candida albicans* (MIC: 75 µM), and the cancer cell line H460 (half maximal inhibitory concentration, IC_50_: 14.97 ± 1.16 µM) (52). Furthermore, PACAP38 exhibited antiviral effects by stimulating the synthesis and release of the β-chemokines CCL3 and CCL5, thereby inhibiting HIV-1 replication (50).

### 3.2 Vasoactive intestinal peptide (VIP)

VIP was initially isolated from the pig duodenum by Said et al. (103). It is a 3.3 kDa polypeptide composed of 28 amino acids (104). The structure of VIPs can change in response to variations in the surrounding environment (105). According to previous studies, approximately 27% of VIPs adopt an α-helix conformation, whereas 27.2% adopt a β-sheet structure, and the remaining 45.8% is characterized as randomly coiled, but it appears to be an α-helix when bound to a lipid or anion (56). VIP is found in two sources within the immune system: terminals present in central and peripheral lymphoid organs and immune cells, mostly lymphocytes. VIP plays an anti-inflammatory role in immune regulation (53).

VIP exerts its antibacterial function through the membrane damage mechanism, although its efficacy against bacteria is more pronounced at a lower NaCl concentration compared to the physiological level of 150 mM (106). Moreover, the diminished antimicrobial activities of VIP in 150 mM NaCl can be restored by the addition of LL-37, suggesting that VIP may exhibit bactericidal effects in conjunction with LL-37 within the physiological milieu of mucosal tissue (107). Notable activity was observed against the gram-negative bacteria *E. coli* (MIC: 1.7 μM) and *P. aeruginosa* (MIC: 1.4 μM), as well as the fungus *C. albicans* (MIC: 15.6 µM) (55). Furthermore, VIP plays a significant role in combating parasites (54). Unlike the membrane damage mechanism commonly induced by AMPs, VIP is initially endocytosed by the parasite, leading to disruption of intracellular lysosome integrity and cytoplasmic glycolytic enzyme function, ultimately resulting in parasite death (54).

### 3.3 α-Melanocyte stimulating hormone (α-MSH)

α-MSH, which was originally discovered in the bovine pituitary gland, is a member of the melanocortin family (61, 108). It is a 13-amino acid polypeptide with a C-terminal amide derived from the enzymatic cleavage of a 36 kD precursor protein known as Proopiomelanocortin (POMC) (109). However, the secondary structure of α-MSH remains unknown. α-MSH is primarily produced by nerve terminals, blood, and various immune cells, such as T_H_2 cells and macrophages, and exhibits potent anti-inflammatory activity (11, 58).

α-MSH is an antimicrobial neuropeptide that exerts its antibacterial effect by inhibiting bacterial adhesion and molecular penetration during the early stages of infection (59), similar to the mechanism of antibiofilm action. It down-regulates the levels of β-1 integrin and HSP70, key molecules involved in *Staphylococcus aureus* invasion of keratinocytes, thereby exhibiting antibacterial activity (59). Moreover, α-MSH effectively permeabilizes the cell membrane of *C. albicans*, resulting in the release of intracellular contents and playing an antifungal role (110). Fjell et al. (60) demonstrated a potent anti-candidiasis effect of α-MSH, leading to its application in the treatment of vulvovaginal candidiasis and chronic respiratory infections, with promising results observed during phase II clinical trials. In terms of antiviral activity, α-MSH suppresses activation of the transcription factor NF-κB, which is known to enhance HIV expression (57).

### 3.4 Orexin-B (ORXB)

The neuropeptide ORXB, consisting of 28 amino acids (64), was initially discovered in a small group of neurons in the hypothalamus and is derived from the hydrolysis of a single precursor protein known as prepro-orexin (111). Orexin-A (ORXA) and ORXB are two isopeptides belonging to the orexin family (112). ORXB is the ancestral form and exhibits significant similarity to VIP in terms of its amphiphilic nature, isoelectric point, and net charge (106). Specifically, ORXB adopts a disordered conformation in aqueous solution and an α-helix structure in a simulated membrane environment (113). It is mainly expressed in neurons located in the lateral and dorsomedial hypothalamus (114) and can regulate the phagocytic function of macrophages in terms of immune regulation (62).

The antimicrobial efficacy of ORXB, similar to that of VIP, was significantly enhanced when it was coexposed to LL-37 at a physiological NaCl concentration of 150 mM (115). This enhancement can be attributed to the interaction between cationic LL-37 and amphiphilic ORXB, which facilitates the binding of ORXB to bacterial membranes and subsequently induces membrane damage (116). ORXB exhibits a broad range of antibacterial activities against both gram-negative bacteria (*E. coli*, *Salmonella typhimurium*, *Klebsiella pneumoniae*) and gram-positive bacteria (*S. aureus*) and demonstrates a strong antibacterial effect at concentrations greater than 25 μg/ml (66). In addition, ORXB has been found to have antiviral activity in inhibiting the infectivity of herpes simplex virus 1 (HSV-1), exceeding the IC_50_ value of 100 μg/mL (66).

### 3.5 Ghrelin

In 1999, Kojima et al. (117) first discovered that Ghrelin is a growth hormone-releasing acylated peptide composed of 28 amino acids secreted by the stomach, and its secondary structure is a putative α-helix (118, 119). Two forms of ghrelin have been identified, namely, acylated ghrelin (AG) and deacylated ghrelin (DAG) (70), with DAG found at higher circulating levels than the AG form (120). Ghrelin is primarily expressed in human peripheral T lymphocytes, B lymphocytes, and neutrophils (121) and exhibits significant anti-inflammatory effects (67).

Ghrelin also has significant antimicrobial effects (70) and exerts its antimicrobial effect primarily through a membrane damage mechanism (122). Moreover, ghrelin concentrations equal to or exceeding 12.5 μg/ml exhibit a significant bactericidal effect against gram-negative bacteria (*E. coli*, *P. aeruginosa*), whereas the bactericidal effects on gram-positive *S. aureus* and *Enterococcus faecalis* are minimal or absent (70). Additionally, ghrelin shows potent antiparasitic activity, such as lysis of parasites by pore formation and plasma membrane disruption, particularly in *African trypanosomes* (68).

### 3.6 Substance P (SP)

In 1931, SP was first identified in the brain and gut of horses by Euler et al. (123), followed by its isolation and subsequent determination of its amino acid sequence in the hypothalamus of cattle (124, 125). SP consists of 11 amino acid residues, and its secondary structure is primarily an α-helix structure (126) It belongs to the tachykinin family. SP is mainly secreted by neurons and can also be produced by inflammatory cells, such as macrophages and dendritic cells, where it promotes the inflammatory response and immune regulation (73).

Moreover, SP has been reported to possess significant antimicrobial activity. Two mechanisms have been identified: one is the acceleration of pathogen virulence factor production when exposed to SP (10 µM), which leads to cytotoxic effects and ultimately pathogen death (127, 128). The other mechanism is the induction of shedding of the S-layer (a barrier against AMPs) of *Bacillus cereus* (129), resulting in damage to cell membrane integrity and exerting its antimicrobial activity (128). SP has demonstrated good antimicrobial activity against the gram-negative bacterium *E. coli* (MIC: 5.7 µg/ml), gram-positive bacterium *Acidophilus* (MIC: 74.1 µg/ml), and fungus *C. albicans* (MIC: 8.1 µg/ml) (55, 115). In addition, SP can competitively bind to the measles virus (MV) receptor and neurokinin-1 receptor, thereby preventing infection of CD46^+^ neurons (130). In the fight against parasites, SP (10^−8^ M) reduces the adherence of *Leishmania brasiliensis* to macrophages, resulting in a repellent chemotactic effect (74).

### 3.7 Adrenomedullin (AM)

The 52-amino acid polypeptide AM belongs to the CGRP family (131, 132). It was initially extracted from adrenal medullary pheochromocytoma by Kitamura et al. (133) in 1993. The secondary structure of AM shows a conserved α-helical region from residues 21 to 33 (optimum antimicrobial activity), whereas the remaining residues do not consistently show ordered regions (42). AM can be synthesized by several immune cells, including macrophages, monocytes and T cells, as well as by lymphoid organs and the gastrointestinal tract (11). Its expression increases under inflammatory conditions, and exerts potent anti-inflammatory effects (11).

AM is also an antimicrobial neuropeptide (78) that functions mainly through a membrane damage mechanism (42). Moreover, AM induces bacterial death by disrupting the peptidoglycan in the cell wall (134). Notably, between 2003 and 2006, Allaker’s research team (42, 78, 135) observed that AM demonstrated equal sensitivity to the gram-negative bacteria *E. coli* (MIC: 0.06 µM), *Haemophilus influenza* (MIC: 2 µM), and the gram-positive bacteria *S. aureus* (MIC: 2 µM) and *S. mutans* (MIC: 2 µM) (78).

### 3.8 Calcitonin gene-related peptide (CGRP)

CGRP, a 37-amino acid neuropeptide, was initially discovered in human medullary carcinoma (81, 136). It belongs to the calcitonin superfamily and adopts an α-helix structure (83). There are two primary forms of CGRP: α-CGRP and β-CGRP. Compared with β-CGRP, α-CGRP is prevalent in both central and peripheral neurons and elicits a stronger immunogenic response (83). CGRP is primarily released from trigeminal ganglia cells and is widely expressed in immune cells, including dendritic cells, T cells, and macrophages (137). It exerts anti-inflammatory effects by modulating innate immune responses (82).

CGRP also has significant antimicrobial effects on various microorganisms found in the skin, respiratory tract, and other anatomical regions (55, 138). CGRP exerts its antimicrobial effect through membrane damage and antibiofilm mechanisms (139, 140), It exhibits considerable efficacy against gram-negative *E. coli* (MIC: 2.1 µg/ml), *P. aeruginosa* (MIC: 5.9 µg/ml), and the fungus *C. albicans* (MIC: 63.1 µg/ml), whereas its bactericidal effects on gram-positive bacteria are minimal (55, 115).

### 3.9 Urocortin-II (UCN II)

Urocortins (UCNs), including UCN I, UCN II, UCN III, Urotensin 1 (found only in fishes), and Sauvagine (found only in amphibians) (141), were initially discovered by Reyes et al. (142) in 2001. UCN II, composed of 38 amino acid residues, has an α-helix structure and possesses amphiphilic properties (89). It belongs to the corticotropin-releasing factor (CRF) family (143). The UCN II can be detected in various types of immune cells, including macrophages/monocytes, T cells, and mast cells, and plays a remarkable anti-inflammatory role in immune responses (144).

UCN II exhibits a broad spectrum of antimicrobial properties (145). Its bactericidal activity is primarily exerted through membrane damage (88, 145). It has good bactericidal effects against gram-negative *E. coli* (median effective concentration, EC_50_: 2.81 µM) and gram-positive bacteria such as *S. mutans* (EC_50_ > 20 µM) and *Micrococcus luteus* (EC_50_: 4.92 µM) (145). In parasites, UCN II destroys promastigotes by forming pores in their membranes, similar to a membrane damage mechanism. The application of UCN II in *L. major*-infected BALB/c mice significantly controls infection (145).

### 3.10 Neuropeptide Y (NPY)

In 1982, NPY was initially isolated from pig brain tissue by Tatemoto et al. (146). It is one of the most abundant neuropeptides in the brain, even more so than VIP (147). NPY belongs to the NPY family and consists of 36 amino acids (148, 149). Its secondary structure is the PP-fold, a generalized α-helix, which comprises a long N-terminal polyproline helix, a type II β-helix, and a long amphiphilic α-helix (150). NPY is ubiquitously present in both the central and peripheral nervous systems, as well as in immune cells such as macrophages, lymphocytes, and neutrophils (151). It acts as an important immunomodulator and plays a significant anti-inflammatory role (92).

NPY also exhibits broad-spectrum antimicrobial effects (152). Its antimicrobial mechanism involves disintegrating the bacterial membrane, leading to lysis and death of pathogenic microorganisms (151, 153). Notably, NPY shows particularly strong antibacterial activity against gram-negative *E. coli* (MIC: 4.2-11 µM), *Aeromonas caviae* (MIC: 14 µM), gram-positive *Nocarida brasiliensis* (MIC: 7 µM), and the fungus *C. albicans* (MIC: 1-2 µM) (115, 152). Furthermore, NPY at a concentration of 10^-9^ M has been shown to exhibit a significant chemoavoidance effect on *L. brasiliensis* parasites (74). Although the protective effects of NPY during retroviral pathogenesis in the central nervous system (CNS), such as in HIV and Ebola, are evident, the complete underlying mechanisms remain unclear (91, 115).

### 3.11 *Hydra* NDA-1

NDA-1, a neuropeptide specific to *Hydra*, was discovered by Augustin et al. (95) in 2017. It consists of 38 amino acids and has a β-sheet secondary structure. NDA-1 is secreted in sensory and ganglion neurons in the ectodermal epithelium, with high expression in the head (hypostome) and foot of *Hydra* (95).

NDA-1 exhibits a broad spectrum of antibacterial activity *in vitro* and can influence the Hydra microbiome, resulting in a lower abundance of *Curvibacter* sp. microbiota in the body column and foot tissue than in the tentacles. The antimicrobial mechanism of NDA-1 may involve interactions between its hydrophobic bag and the bacterial membrane, similar to the membrane damage mechanism (95). It demonstrates high toxicity against the gram-negative bacteria *Curvibacter* sp. (MIC: 0.4 µM), *Acinetobacter* sp. (MIC: 7 µM), and *E. coli* (MIC > 14 µM), as well as the gram-positive bacteria *Bacillus megaterium* (MIC: 0.4 µM), *Trichococcus pasteurii* (MIC: 0.9 µM), and *Trichococcus collinsii* (MIC: 0.4 µM) (95).

### 3.12 Catestatin (CST)

In 1997, Mahata et al. (96) first discovered and identified CST as a catecholamine release inhibitory peptide consisting of 21 amino acids. CST has a highly alkaline nature with an amphiphilic conformation (97) and adopts a β-sheet secondary structure (154). Furthermore, CST is primarily expressed in peripheral mononuclear cells, mast cells, and macrophages and exerts its anti-inflammatory effect on immune regulation (98, 155).

The antimicrobial activity of CST was initially demonstrated by Briolat et al. (156) in 2005. The antimicrobial mechanism of CST is similar to that of typical AMPs, as CST has been shown to cause membrane damage (99). Notably, CST exhibits significant antimicrobial efficacy against various pathogens, including the gram-negative bacteria *E. coli* (MIC: 15 µM) and *P. aeruginosa* (MIC: 50 µM), the gram-positive bacterium *M. luteus* (MIC: 5 µM) and Group A *Streptococcus* (MIC: 75 µM), and the filamentous fungus *Aspergillus fumigatus* (MIC: 80 µM) (156, 157).

## 4 Therapeutic potential in brain infectious disease

### 4.1 Macrophages act as bacterial carriers to break through the BBB

The BBB serves as a highly regulated interface between the bloodstream and the brain, playing a crucial role in the CNS by facilitating infection signaling to the brain (158). During infections and autoimmune diseases, macrophages can infiltrate the brain to eliminate pathogens, such as Group B *Streptococcus* (GBS) infection of the CNS, which triggers the recruitment of macrophages through immune deficiency (Imd) (159). In recent years, it has been discovered that macrophages serve as replicative niches for various bacteria, such as *P. aeruginosa*, *E. coli*, *Yersinia pestis*, Group A *Streptococcus*, and GBS (160). These macrophages can act as splenic reservoirs of sepsis and facilitate the survival and replication of *Streptococcus pneumoniae* within the intracellular environment. In other words, macrophages function as bacterial carriers, akin to “Trojan horses,” enabling successful traversal of the BBB and subsequent brain infection (160).

Bacteria must overcome diverse antimicrobial stimuli to survive within macrophages (160). Metal toxicity represents a prominent mechanism employed by macrophages for bacterial eradication (161). Korir et al. (160) demonstrated that GBS strains possess virulence mechanisms that enable prolonged survival within macrophages. GBS cells express *cadD*, which encodes a crucial metal efflux transporter that helps remove excess metal ions from the cell, thereby conferring resistance to metal toxicity (160). Apart from the *cadD* locus, *sczA* and *czcD* have also been identified as being involved in metal efflux (162). Moreover, *cadD* orthologues have been detected in other pathogens, such as *S. aureus* (160). In general, when pathogens enter macrophages, they evade intracellular metal toxicity through metal efflux, thereby enabling prolonged survival (160). Additionally, studies have also revealed that alterations in macrophage polarization are partially attributed to variations in macrophage stimulation during different *S. aureus* infection scenarios (163). M1 (pro-inflammatory) or M2 (anti-inflammatory) polarization leads to different responses of macrophages to *S. aureus*. M1 polarization resulted in bacterial death through reactive oxygen species (ROS), acidic pH, enzyme nutrient restriction and AMPs (164, 165). However, under certain circumstances, *S. aureus* can manipulate macrophage autophagy to induce M2 polarized, and effectively evade and manipulate macrophages, ultimately hindering macrophage recruitment, phagocytosis and degrative abilities (163).

### 4.2 Antimicrobial neuropeptides are upregulated during pathogen infection

Numerous neuropeptides share similarities with AMPs in terms of their size, hydrophobicity, charge, and amino acid composition (55). These neuropeptides, which are involved in neurological or neuroendocrine signaling processes, have shown a wide range of antimicrobial effects against various microorganisms (55). Generally, the upregulation of neuropeptide expression is often observed in response to bacterial-induced inflammatory conditions, such as pulpitis, periodontal disease, and *in vivo* bacteremia (55, 166). Lundy’s research team (166–168) discovered that in the odontoblastic and subodontoplastic layers of the dental pulp close to caries lesions, the sprouting of peptidergic nerves leads to increased levels of neuropeptides (such as VIP, NPY, and SP) at the site of local inflammation, thereby facilitating their direct antimicrobial actions. Furthermore, Lee et al. (1) reported a significant upregulation of PACAP expression—up to a 50-fold increase—in response to infection with *S. aureus* or *C. albicans*, suggesting that PACAP is involved in the antimicrobial defense of the CNS by preventing the infiltration of inflammatory cells.

### 4.3 Anti-inflammatory activity of AMPs

For a substantial period, the neuroendocrine and immune systems are considered two separate networks that regulate the balance between the host and its surroundings (169). The neuroendocrine system responds when stimulated by external environmental factors, whereas the immune system assumes its role in fighting invading bacteria, viruses, and other pathogens (169). However, in the past three decades, significant progress in research on the immune system and neuroendocrine system has established a complex and profound interplay between these two systems (170). On the one hand, the neuroendocrine system modulates immune responses through the release of hypothalamic and pituitary hormones, as well as the activation of the autonomic nervous system (171). On the other hand, the immune system detects stimuli that go unnoticed by the neuroendocrine system, such as bacteria, viruses, and tumors, and converts them into signals that prompt a response from the neuroendocrine system, helping to regulate fever and sleep problems (169). This bidirectional communication mechanism between these two entities plays a critical role in perceiving external stimuli, maintaining homeostasis, orchestrating immune responses, and governing growth and development (172). The underlying mechanism can be attributed to a shared biochemical language among them, including common neurotransmitters (such as neuropeptides and hormones), immune cytokines, and other ligands, along with their respective receptors (173). The complex interplay between the neuroendocrine system and the immune system plays a pivotal role in eradicating pathogens and restoring immune homeostasis (13). However, when this delicate equilibrium is disrupted, it can trigger a cascade of detrimental effects on infectious and autoimmune diseases, exerting a profound impact on pathological processes (174).

The induction of immune tolerance is crucial for maintaining immune homeostasis, regulating autologous reactive T cells, preventing the development of autoimmune diseases, and achieving transplantation tolerance (175). Inflammation is an essential process for pathogen eradication; however, uncontrolled inflammation, especially in the brain, can lead to severe adverse effects on the host. Therefore, the investigation of endogenous factors that regulate immune tolerance and inflammation represents a crucial research topic within the field of immunology.

Between 2000 and 2008, Delgado’s team made the groundbreaking discovery that neuropeptides secreted by immune cells exert inhibitory effects on inflammation while maintaining immune homeostasis. These neuropeptides mainly include VIP, α-MSH, UCN I, AM, and cortistatin (11, 13, 169). Among the antibacterial neuropeptides mentioned in this paper, the main ones with anti-inflammatory activity include PACAP (176), VIP (11), α-MSH (11), Ghrelin (177), AM (11), NPY (178), UCN II (179), CGRP (180), and CST (98). The mechanism of action can be summarized as follows: antimicrobial neuropeptides exert their effects on macrophages, monocytes, and microglia through regulatory T cells, leading to the inhibition of the production and release of inflammatory factors (TNF-α, IL-6, and IL-1β), chemokines (CCL5, IL-8, and IP-10), and NO. Additionally, they promote the production of anti-inflammatory cytokines such as TGFβ, which exerts their anti-inflammatory effects (181). Moreover, antibacterial neuropeptides play a pivotal role in maintaining the equilibrium between T_H_2 and regulatory T cells as well as between T_H_1 cells within the body, thereby ensuring a state of homeostasis between anti-inflammatory and proinflammatory factors to prevent the onset of autoimmune diseases (11).

### 4.4 Is the antimicrobial and anti-inflammatory activity of antimicrobial neuropeptides a defense mechanism of the brain?

The mechanism by which the vertebrate brain defends against pathogen infection is currently a key research area. Inflammatory attacks, facilitated by immune cells, can cause damage, and whereas the brain has limited ability to repair itself, so pathogenic microorganisms must be eliminated with minimal collateral damage to the organ itself (1, 2). Thus, it is likely that the brain possesses an immune defense mechanism that has yet to be fully understood. Through an extensive review of the literature, it has been observed that certain neuropeptides synthesized by nerve cells or immune cells share significant similarities with AMPs in terms of their physicochemical properties. These neuropeptides have demonstrated a wide range of antimicrobial activity *in vitro*, effectively guarding nerve tissue against microbial invasion (1). Furthermore, these antimicrobial neuropeptides have also been demonstrated potent anti-inflammatory properties and can contribute to the regulation of immune tolerance in various immune disorders (11). Notably, the connection between this anti-inflammatory activity and the brain’s defense against pathogen infection remains poorly explored. Therefore, we proposed a hypothesis that the antimicrobial activity of neuropeptides can efficiently eliminate pathogenic microorganisms in the brain, whereas their anti-inflammatory activity can suppress the occurrence of off-target inflammation. Further investigations of this topic will be a primary focus of future research. Additionally, neuropeptides possessing both antimicrobial and anti-inflammatory properties may hold great potential as a novel class of antimicrobial drugs. Extensive pharmaceutical research and clinical testing may prove valuable in the treatment of bacterial meningoencephalitis. If successfully developed into a pharmaceutical agent, this approach could offer a significant breakthrough in the treatment of brain infectious diseases, particularly in light of concerns regarding the misuse of antibiotics.

## 5 Conclusion

Antimicrobial neuropeptides protecting vertebrate brains against infection is a newly discovered brain defense mechanism in 2021 (1). However, given the limited regenerative capacity of the brain in response to immune cell-mediated inflammatory attacks, the presence of an unexplored layer of immune defense mechanisms has become increasingly significant.

In this review, we have provided a concise summary of the physicochemical characteristics and potential antimicrobial mechanisms of AMPs. Several neuropeptides, which are produced by nerve cells or immune cells, are remarkably similar to AMPs and exhibit a broad spectrum of antimicrobial activities. Subsequently, we have undertaken an extensive review of 12 previously documented neuropeptides that possess antimicrobial properties. Our comprehensive analysis included an exploration of their origin, structural attributes, possible antimicrobial mechanisms, and observed efficacy against microbial agents. Furthermore, it has been noted that a majority of these antimicrobial neuropeptides (9 out of 12) also exhibit potent anti-inflammatory activity, indicating their potential involvement in regulating immune disorders. Consequently, the combined antimicrobial and anti-inflammatory activities of neuropeptides could play a pivotal role in fortifying the defense mechanisms of the brain against pathogenic invaders. Moving forward, we anticipate the validation of this hypothesis in future research endeavors.
